# Vibrations of a piezoelectric Timoshenko beam with resistive-inductive electrodes

**DOI:** 10.1007/s11012-025-02010-5

**Published:** 2025-07-14

**Authors:** Juergen Schoeftner

**Affiliations:** Institute of Technical Mechanics, Johannes Kepler University Linz, Altenberger Strasse 69, Linz, 4040 Upper Austria Austria

**Keywords:** Piezoelectric effect, Resistive electrodes, Linear piezoelectric beam modeling, Passive vibration control, Piezoelectric Timoshenko beam, Bending vibrations

## Abstract

This paper presents a one-dimensional theory for moderately thick piezoelectric beam-type structures with imperfect resistive electrodes. For practical applications, a special goal is also the finite element discretization of the electromechanically coupled partial differential equations, which combine the Telegrapher’s equations with the elastic properties of a Timoshenko beam. Unlike ideal electrodes, which satisfy the equipotential area condition, the voltage distribution in resistive electrodes is governed by the diffusion equation. For the electrical domain, Kirchhoff’s voltage and current rules are applied to derive the parabolic differential equation, which is driven by the time derivative of the axial strain. It is demonstrated that the current flow through the electrodes of the piezoelectric layer depends on the electrode resistance and the capacitance. For the mechanical domain, d’Alembert’s principle is combined with the piezoelectric constitutive equations to derive an extended version of the Timoshenko beam equations, incorporating the *x*-dependent voltage drop across the electrodes. A one-dimensional finite element is then formulated using Timoshenko shape functions for the deflection and the rotation angle, along with linear shape functions for the voltage drop along the beam segment. For the validation of the model a clamped-hinged piezoelectric beam is used as a benchmark example to compare the results of the one-dimensional discretization with two-dimensional finite element (FE) simulations. Various types of resistive electrodes are considered, including static deflections, dynamic vibrations, and eigenfrequency analyses. The results demonstrate that the derived piezoelectric beam model also includes the case of ideal electrodes (short- and open-circuited), when the sheet resistance is very low, and the case of a non-electroded piezoelectric beam, when the sheet resistance is very high.

## Introduction

Intelligent structures are systems composed of multifunctional materials that control motion in a targeted manner. One way to influence the structure is through the piezoelectric effect either via feedforward or feedback control of piezoelectric actuators or through the dissipation of electrical energy for passive control. An overview of the research field of adaptronics, where the piezoelectric effect is an important mechanism for measuring physical quantities or controlling structures, is provided by Janocha [1]. Classical and introductory works on piezoelectricity include those by Crawley [2], Chopra [3], and Tzou [4]. For the specific application of piezoelectric transducers or patches, readers are referred to the books by Moheimani and Fleming [5] and Preumont [6], which focus primarily on the reduction of structural vibrations.

The concept of passive vibration control dates back to Forward [7], who was the first to attenuate vibrations in an optical device by connecting a piezoelectric transducer to an external circuit. This circuit was later replaced by resistive-inductive impedances by Hagood and Flotow [8], which paved the way for single- and multi-mode shunt strategies as well as semi-passive control methods, such as those proposed by Niederberger et al. [9], Park [10], and Trindade and Maio [11]. The experimental results of a shunt-damped cantilevered plate obtained by Hagood and Flotow [8] were used by Thornburgh and Chattopadhyay [12] to validate their finite element (FE) model. Unlike most electromechanically coupled FE formulations, they used the electrical displacement field instead of the electric potential as the electrical degree of freedom (DOF). Later, they applied this formulation to develop an optimization procedure for calculating the parameters of an attached electrical circuit for a plate, as demonstrated by Thornburgh and Chattopadhyay [13].

Krommer and Irschik [14] investigated the influence of the electric field on the transverse vibrations of a piezoelectric bimorph. Later Krommer [15] developed a beam model that effectively incorporates both direct and indirect piezoelectric effects within the framework of the Bernoulli-Euler beam theory. He also examined the impact of short-circuited, open-circuited, and non-electroded configurations on deflection and eigenfrequencies. An extension to the Timoshenko beam theory was later provided by Krommer and Irschik [16]. A piezoelectric beam theory for moderately thick structures, where the electrodes of the piezoelectric layers are connected to external electric circuits, was introduced by Schoeftner and Irschik [17]. This model was subsequently used to derive conditions for passive shape control, as described in Schoeftner and Irschik [18]. The study confirmed that both the optimal width of the piezoelectric layers and the impedance of the electric circuit must be properly tuned to completely reduce time-harmonic vibrations caused by external loads.

Finite element beam formulations for piezoelectric sandwich structures were developed by Benjeddou et al. [19], [20]. Based on a variational formulation of a sandwich beam, the model accounts for both extension and shear actuation mechanisms and is expressed in terms of mass and stiffness matrices as well as mechanical and electrical force vectors. Furthermore, static and dynamic analyses were conducted for sandwich structures with both large and small soft cores, and the results were compared with existing literature.

Unlike metal electrodes, polymer electrodes cause a significant potential loss across the electrode surface. This phenomenon is exploited in position-sensitive touchpads to detect the location of a pressure source, as demonstrated by Buchberger et al. [21, 22]. The first model to properly couple mechanical assumptions, the piezoelectric effect, and resistive electrodes was introduced by Lediaev [23], who analyzed the interaction of moderately and low-conductive electrodes with three-dimensional (3D) vibrations of a cantilever.

Buchberger and Schoeftner [24] integrated elementary beam theory, piezoelectricity, and resistive electrodes to derive an extended beam differential equation, which is coupled with a diffusion equation describing the electric potential of the electrodes. It has been demonstrated that shape control can be achieved by appropriately tuning the electrode resistance, as shown by Schoeftner et al. [25]. The practical application of this innovative control method has been validated through a granted patent (Schoeftner and Buchberger [26]) and its experimental implementation (Schoeftner et al. [27]).

The goal of this contribution is manifold: On the one hand, this contribution considers Timoshenko kinematics and resistive-inductive electrodes and thus can be considered as an extension of [24, 28] and [29], where a Bernoulli-Euler beam is considered. On the other hand, it is shown how to discretize the electromechanically coupled differential equation by means of finite elements. The partial differential equations for a piezoelectric bimorph with resistive-inductive electrodes are first revisited, considering the coupling between axial and lateral deflections. For a symmetric bimorph, the axial motion can be neglected, resulting in a piezoelectrically extended version of the Timoshenko equations, which are coupled to the Telegrapher’s equations (second-order in both space and time), if resistive-inductive electrodes are considered. If, however, the inductive property of the electrodes is disregarded, the Timoshenko equations are coupled to the diffusion equation for the voltage (second-order in both space and first-order in time). These electromechanically coupled equations are then discretized: Timoshenko ansatz functions are used for the deflection and the rotation, and linear ansatz functions are used for the voltage distribution to derive a one-dimensional finite element. A clamped-hinged piezoelectric bimorph is used for validation: First, a natural frequency and a quasi-static deflection analysis are conducted for a thin and a moderately thick beam under a uniformly distributed load. From the electrical point of view, perfect electrodes with short- and open-circuit conditions and poorly conductive electrodes (non-electroded conditions) are considered. Finally, a frequency response analysis close to the first eigenfrequency is performed to minimize the harmonic deflection. Resistive and inductive properties are optimized in order to highlight the practical significance of optimally-tuned resistive-inductive electrodes and terminal impedances.

## Modeling of a moderately thick piezoelectric beam with resistive electrodes – basic relations

The constitutive relations are the material laws which relate mechanical (the axial stress $\sigma _{xx}$ and the shear stress $\sigma _{xz}$ and the corresponding strains $\varepsilon _{xx}$, $\gamma _{xz}$) and electrical variables (the *z*-component of the electric displacement ${D}_z$ and the electric field ${E}_z$). The coupled mechanical and electrical constitutive relations can be written as

1 $$\begin{aligned} {\sigma }^k_{xx} = \tilde{C}^k_{11} {\varepsilon }^k_{xx} - \tilde{e}^k_{31} {E}^k_z \nonumber \\ \sigma ^k_{xz} = \tilde{C}^k_{55} \gamma ^k_{xz} - \tilde{e}^k_{15} E_x^k \nonumber \\ {D}^k_z = \tilde{e}^k_{31} {\varepsilon }^k_{xx} + \tilde{\kappa }^k_{33} {E}^k_z. \end{aligned}$$

The material properties $\tilde{C}^k_{11}$, $\tilde{C}^k_{55}$, $\tilde{e}^k_{31}$, $\tilde{e}^k_{15}$ and $\tilde{\kappa }^k_{33}$ denote the effective values of the elastic and the piezoelectric modulus and the strain-free permittivity, which are derived from the tensor components of the material parameters under the beam assumptions, see Appendix A. The tracer *k* is the indicator for the $k^{th}$ piezoelectric layer. The distances to the *x*-axis are denoted as $z_{2k}$ and $z_{1k}$, so the height of a layer is given by $h_{k} =z_{2k} - z_{1k}$. The electric field in the thickness direction is equal to the negative spatial derivative of the electric potential with respect to the thickness coordinate

2 $$\begin{aligned} E_{z}^k = -\varphi _{,z}^k \approx -\frac{V^k}{h_k} \rightarrow \;\varphi\; (x,z) = \varphi (x,z_{1k}) + V^k(x) \frac{z}{h_k} \end{aligned}$$

Eq. (2) neglects the influence of the deformation on the electric fields, when the induced electric potential is properly calculated based on the kinematic assumption and by means of Gauss’ law of electrostatics. One observes that the potential drop $$V^k(x)$$ is a linear function in the thickness direction and depends on the surface potentials $$\varphi (x,z_{1k})$$ and $$\varphi (x,z_{2k})$$ and on the layer thickness $$h_k = z_{2k} - z_{1k} $$. Considering bending vibrations, the influence of $$E_x^k$$ is much lower than the *z*-component of the electric field and is usually neglected

3 $$\begin{aligned} E_{x}^k \approx 0 \end{aligned}$$

The horizontal and the transverse displacements along the *x*-axis are denoted by $u_0(x,t)$ and $w_0(x,t)$, and the rotation angle is denoted by $\psi$. Assuming Timoshenko theory, the displacement field as a function of *x* and *z* is given by

4 $$\begin{aligned} u(x,z,t) = u_0(x,t) + z \psi (x,t) \nonumber \\ w(x,z,t) = w_0(x,t) \end{aligned}$$

This approach (4) yields for the strains

5 $$\begin{aligned} \varepsilon _{xx} = u_{0,x} + z \psi _{,x} \nonumber \\ \gamma _{xz} = \psi + w_{0,x} \end{aligned}$$

### Electrical equations

**Fig. 1:**
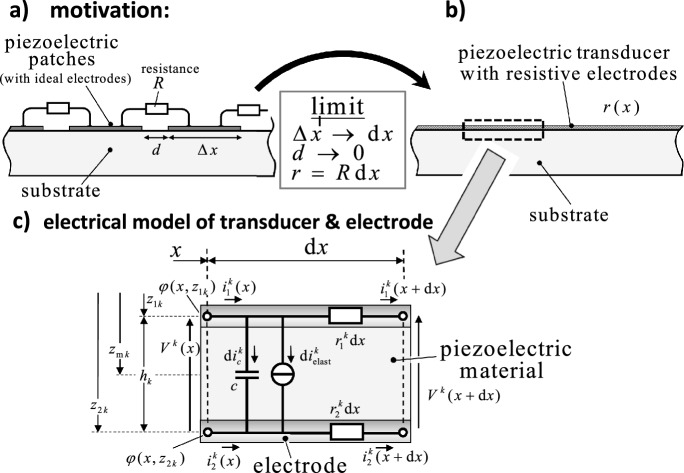
Motivation for a beam model with resistive electrodes: (**a**) A beam with piezoelectric patches connected by resistances is the discrete model of a piezoelectric beam with resistive electrodes, (**b**) equivalent electrical model of a piezoelectric segment with length $\textrm{d}x$

Figure 1c shows the equivalent model of the piezoelectric layer and the resistive electrodes. Both surfaces are assumed to be covered by finitely conductive electrodes, i.e. the equipotential area condition is not fulfilled because the current flow $i_1^k$, $i_2^k$ over the surfaces reduces the potential in the flow direction due to the resistance per unit length $r_1$ and $r_2$. Hence, the potential drop is a function of *x* and is not constant $V^k(x) \ne \mathrm {const.}$

Inserting (5) into the third relation (1) one finds for the electric displacement

6 $$\begin{aligned} D_{z}^k = \tilde{e}^k_{31} \left( u_{0,x} + z \psi _{,x} \right) - \tilde{\kappa }^k_{33} \varphi _{,z} \end{aligned}$$

For our one-dimensional theory, Gauss law of electrostatics states that the divergence of the electric displacement vector vanishes $$\nabla \cdot D = 0$$. Because $$D_{x}^k $$ is disregarded, the electric displacement is constant in the *z*-direction because of $$D_{z,z}^k = 0$$.

This means that the electric displacement $D_{z}$ does not depend on *z* and therefore its mean value in the thickness direction equals $D_{z}$. Eq. (6) can be reformulated as

7 $$\begin{aligned} D_{z}^k = \frac{1}{h_k} \int _{z_{1k}}^{z_{2k}} \tilde{e}^k_{31} \left( u_{0,x} + z \psi _{,x} \right) - \tilde{\kappa }^k_{33} \varphi _{,z} \, \textrm{d}z = \tilde{e}^k_{31} \left( \frac{ z_{2k} + z_{1k} }{ 2 } \psi _{,x} + u_{0,x} \right) - \frac{ \tilde{\kappa }^k_{33} }{h_k} V^k \end{aligned}$$

One finds the electric field by inserting Eq. (7) into Eq. (1)

8 $$\begin{aligned} E_{z}^k = - \frac{ V^k }{h_k} + \frac{ \tilde{e}^k_{31} }{ \tilde{\kappa }^k_{33} } \left( \frac{ z_{2k} + z_{1k} }{ 2 } - z \right) \psi _{,x} \end{aligned}$$

Finally, the electric potential is obtained by integrating Eq. (8) with respect to *z*, and prescribing either the potential at $z=z_{1k}$ or $z=z_{2k}$

9 $$\begin{aligned} \varphi (x,z) = \varphi (x,z_{1k}) + \frac{ V^k }{h_k} z + \underbrace{ \frac{ \tilde{e}^k_{31} }{ 2 \tilde{\kappa }^k_{33} } \left( z - z_{2k} \right) \left( z - z_{1k} \right) \psi _{,x} }_{ \mathrm {induced \,\, electric \,\, potential} } \end{aligned}$$

The last term in Eq. (9) describes the small influence of the bending deformation on the electric potential. This higher-order effect of the induced potential may be neglected, so that only the linear term remains in Eq. (9). Hence, the relation on the right-hand side of Eq. (2) is a good approximation for most practical configurations.

As indicated in Figure 1c, the total leakage flow $\textrm{d}i^k_D(x,t)$ per unit length $\textrm{d}x$ (from the upper to the lower electrode) consists of an elastic part $\textrm{d}i^k_{\textrm{elast}}$ and of an capacitive part $\textrm{d}i^k_{c}$. The time-derivative of Eq. (6) multiplied by the layer width $b_k$ and the infinitesimal length $\textrm{d}x$ is equal to this leakage flow, hence it follows

10 $$\begin{aligned} \textrm{d}i^k_D(x,t) = \dot{D}^k_z(x,t) b_{k}(x) \, \textrm{d}x = \textrm{d}i^k_{\textrm{elast}}(x,t) + \textrm{d}i^k_{c}(x,t). \end{aligned}$$

Inserting Eq. (7) into Eq. (10) one obtains for the total leakage flow between the upper and the lower electrode

11 $$\begin{aligned} i^k_D = \tilde{e}^k_{31} b_{k} \left( \frac{ z_{2k} + z_{1k} }{ 2 } \dot{\psi }_{,x} + \dot{u}_{0,x} \right) - \frac{ \tilde{\kappa }^k_{33} b_{k} }{h_k} \dot{V}^k = P^k_{M} \dot{\psi }_{,x} + P^k_{N} \dot{u}_{0,x} - c^k \dot{V}^k \end{aligned}$$

The capacitance per unit length $c^k$ and the piezoelectric coupling constants $P^k_{M}$, $P^k_{N}$ are introduced in Eq. (11)

12 $$\begin{aligned} c^k = \tilde{\kappa }^k_{33} b_{k} / h_k \end{aligned}$$

13 $$\begin{aligned} P^k_{M} = \tilde{e}^k_{31} b_{k} \left( z_{2k} + z_{1k} \right) / 2 \end{aligned}$$

14 $$\begin{aligned} P^k_{N} = \tilde{e}^k_{31} b_{k} \end{aligned}$$

In the block diagram model (Figure 1c) the leakage flow $i^k_D$ causes a reduction of flow over the internal electrode, while causing an increase over the external electrodes. It follows with Eq. (11)

15 $$\begin{aligned} i^k_{1,x} = - i^k_{D,x} = - P^k_{M} \dot{\psi }_{,x} - P^k_{N} \dot{u}_{0,x} + c^k \dot{V}^k \nonumber \\ i^k_{2,x} = i^k_{D,x} = P^k_{M} \dot{\psi }_{,x} + P^k_{N} \dot{u}_{0,x} - c^k \dot{V}^k \end{aligned}$$

If one applies Kirchhoff’s voltage rule for both electrodes, the voltage drop is caused by $i^k_{2} r^k_2$ (lower layer) and $i^k_{1} r^k_1$ (upper layer), one finds

16 $$\begin{aligned} \varphi ^k (x+\textrm{d}x,z_{2 k}) = \varphi ^k (x,z_{2 k}) - i^k_{2} r^k_2 \, \textrm{d}x \rightarrow \varphi ^k_{,x} (x,z_{2 k}) = - i^k_{2} r^k_2 \nonumber \\ \varphi ^k (x+\textrm{d}x,z_{1 k}) = \varphi ^k (x,z_{1 k}) - i^k_{1} r^k_1 \, \textrm{d}x \rightarrow \varphi ^k_{,x} (x,z_{1 k}) = - i^k_{1} r^k_1 \end{aligned}$$

Combining Eq. (15) and taking the partial derivative of Eq. (16) with respect to *x*, one obtains

17 $$\begin{aligned} \varphi ^k_{,xx} (x,z_{2 k}) - \varphi ^k_{,xx} (x,z_{1 k}) = c^k \left( r_1^k + r_2^k \right) \dot{ V}^k - \left( r_1^k + r_2^k \right) \left( P^k_{M} \dot{\psi }_{,x} + P^k_{N} \dot{u}_{0,x} \right) \end{aligned}$$

Finally, using the relation between electric potentials and the voltage drop across the electrodes $\varphi ^k (x,z_{2 k}) - \varphi ^k (x,z_{1 k}) = V^k$, see Eq. (2), one derives the parabolic partial differential equation from (17)

18 $$\begin{aligned} -V^k_{,xx} + c^k \left( r_1^k + r_2^k \right) \dot{ V}^k = \left( r_1^k + r_2^k \right) \left( P^k_{M} \dot{\psi }_{,x} + P^k_{N} \dot{u}_{0,x} \right) \end{aligned}$$

where the left-hand side is identical to the heat equation if the electric voltage $V^k$ is replaced by the temperature *T*. Similar to thermoelastic problems, the right-hand side of Eq. (18) is the driving term and depends on the strain rate. It is noted that Eq. (18) is a special case of the Telegrapher’s equation when inductive properties of the electrodes are neglected: Considering also the inductance per unit length of the electrodes $l_1^k$, $l_2^k$, which causes an additional voltage drop of the electrodes, one finds in a similar manner

19 $$\begin{aligned} -V^k_{,xx} + c^k \left( r_1^k + r_2^k \right) \dot{ V}^k + c^k \left( l_1^k + l_2^k \right) \ddot{ V}^k = \left( r_1^k + r_2^k \right) P^k_{M} \dot{\psi }_{,x} + \left( l_1^k + l_2^k \right) P^k_{M} \ddot{\psi }_{,x} \nonumber \\ \left( r_1^k + r_2^k \right) P^k_{N} \dot{u}_{0,x} + \left( l_1^k + l_2^k \right) P^k_{N} \ddot{u}_{0,x} \end{aligned}$$

This form of the telegrapher’s equation is a damped wave equation: The wave character is due to the inductive electrodes $l^k$, which is related to the second time-derivative of the voltage drop $\ddot{ V}^k$. The damping character is caused by the resistive electrodes $r^k$ and the first time-derivative of the voltage $\dot{ V}^k$.

### Mechanical equations

In order to obtain the governing mechanical equations of the smart composite beam, D’Alembert’s principle in the formulation of Lagrange is utilized, cf. Ziegler [30]

20 $$\begin{aligned} \int _V \rho \ddot{u}_i \delta u_i \, \textrm{d}V + \int _V \sigma _{ij} \delta \varepsilon _{ij} \, \textrm{d}V = \int _S p_i \delta u_i \, \textrm{d}S \end{aligned}$$

In Eq. (20) $\sigma _{ij}$ and $\varepsilon _{ij}$ are stress and strain tensors, $u_i$ is the vector of three displacements and $p_i$ denotes the surface traction vector. The virtual displacement is indicated by $\delta u_i$. *V* and *S* are the volume and surface of the beam. According to our kinematic assumption (4), we neglect warping effects and that the vertical deflection and the rotation angle of each layer is the same. Hence, the outcome of our equations is an equivalent single layer theory considering the effect of shear and the influence of the piezoelectric layers and the resistive electrodes. The first part in Eq. (20) represents the time-derivation of the kinetic energy. Inserting the kinematic assumptions (4), one finds for the inertial terms

21 $$\begin{aligned} \int _V \rho \ddot{u}_i \delta u_i \, \textrm{d}V = \int _V \rho \left( \ddot{u}_0 + z \ddot{\psi } \right) \left( \delta {u}_0 + z \delta \psi \right) + \rho \ddot{w}_0 \delta {w}_0 \, \textrm{d}V = \nonumber \\ \int _0^l \left[ \left( B_{uu} \ddot{u}_0 + B_{u \psi } \ddot{\psi } \right) \delta {u}_0 + \left( B_{u \psi } \ddot{u}_0 + B_{\psi \psi } \ddot{\psi } \right) \delta {\psi } + B_{ww} \ddot{w}_0 \delta {w}_0 \right] \, \textrm{d}x \end{aligned}$$

where the mass moments of inertia are

22 $$\begin{aligned} \left( B_{uu} = B_{ww}, B_{u \psi }, B_{\psi \psi } \right) = \int _A \rho \left( 1, \, z,\, z^2 \right) \, \textrm{d}A \nonumber \\ = \sum _{k=1}^K \rho ^k b_k \left( z_{2k} - z_{1k}, \frac{ z_{2k}^2 - z_{1k}^2 }{ 2 }, \frac{ z_{2k}^3 - z_{1k}^3 }{ 3 } \right) \end{aligned}$$

For the strain energy only the axial stress $\sigma _{xx}$ and the shear stress $\sigma _{xz}$ are considered, while the other components are neglected. Taking advantage of the exact relation of the electric fields, see Eq. (8), it follows for the axial and the shear stress

23 $$\begin{aligned} {\sigma }^k_{xx} = \tilde{C}^k_{11} u_{0,x} + \left[ \tilde{C}^k_{11} z - \frac{ (\tilde{e}^k_{31})^2 }{ \tilde{\kappa }^k_{33} } \left( \frac{ z_{2k} + z_{1k} }{ 2 } - z \right) \right] \psi _,{x} + \frac{ \tilde{e}^k_{31} }{ h_k } {V}^k_z \end{aligned}$$

24 $$\begin{aligned} \sigma ^k_{xz} = \tilde{C}^k_{55} \left( \psi + w_{0,x} \right) \end{aligned}$$

Equation 23 requires some explanation: First, one observes that the last term within the square brackets is due to the induced electric potential, see Eq. (8) or (9), which influences the bending deflection $\psi$ (but not the axial deflection $u_0$). Second, the equivalent single layer (ESL) theory is an appropriate model for multi-layer beams only if the elastic moduli of the layer materials are in the same range. A recent study by the author [32] showed that for sandwich structures where the Young’s modulus ratio of core layer to the face sheets is smaller than 1/10, more advanced beam theories should be considered.

Considering the axial strain relation $\varepsilon _{xx} = u_{,x}$ one finds

25 $$\begin{aligned} \int _V \sigma _{xx} \delta u_{,x} \, \textrm{d}V = -\int _V \sigma _{xx,x} \delta u \, \textrm{d}V + \int _A \sigma _{xx} \delta u \, \textrm{d}A |_{x=0}^l = \nonumber \\ -\int _0^l N_{,x} \delta u_{0} \, \textrm{d}x -\int _0^l M_{,x} \delta \psi \, \textrm{d}x + N \delta u_0 |_{x=0}^l + M \delta \psi |_{x=0}^l \end{aligned}$$

Proceeding in the same way and taking advantage of the shear strain $\gamma _{xz} = \psi + w_{0,x}$, one finds

26 $$\begin{aligned} \int _V \sigma _{xz} \delta \gamma _{xz} \, \textrm{d}V = \int _V \sigma _{xz} \left( \delta \psi + \delta w_{0,x} \right) \, \textrm{d}V = \int _0^l Q \delta \psi \, \textrm{d}x - \int _0^l Q_{,x} \delta w_0 \, \textrm{d}x + Q \delta w_0 |_{x=0}^l \end{aligned}$$

The normal force *N*, the bending moment *M* and the shear force *Q* appear in Eqs.(25) and (26). One finds by considering Eq. (1)

27 $$\begin{aligned} N(x) = \sum _{k=1}^K \int _{z_{1k}}^{z_{2k}} \sigma _{xx}^k b_k \, \textrm{d}z = K_{N} u_{0,x} + K_{NM} \psi _{,x} + \sum _{k=1}^K P^k_{N} V^k(x) \end{aligned}$$

28 $$\begin{aligned} M(x) = \sum _{k=1}^K \int _{z_{1k}}^{z_{2k}} \sigma _{xx}^k b_k z\, \textrm{d}z = K_{NM} u_{0,x} + K_M \psi _{,x} + \sum _{k=1}^K P^k_{M} V^k(x) \end{aligned}$$

29 $$\begin{aligned} Q(x) = \sum _{k=1}^K \int _{z_{1k}}^{z_{2k}} \sigma _{xz}^k b_k \, \textrm{d}z = K_Q \left( \psi + w_{0,x} \right) \end{aligned}$$

The axial, the bending and the shear stiffness are denoted by $K_N$, $K_M$ and $K_Q$

30 $$\begin{aligned} K_{N} = \sum _{k=1}^K \tilde{C}_{11}^k b_k \left( z_{2k} - z_{1k} \right) \end{aligned}$$

31 $$\begin{aligned} K_{NM} = \sum _{k=1}^K \tilde{C}_{11}^k b_k \frac{ z_{2k}^2 - z_{1k}^2 }{ 2 } \end{aligned}$$

32 $$\begin{aligned} K_{M} = \sum _{k=1}^K \left[ \tilde{C}_{11}^k b_k \frac{ z_{2k}^3 - z_{1k}^3 }{ 3 } + \frac{ (\tilde{e}^k_{31})^2 }{ 12 \tilde{\kappa }^k_{33} } \left( z_{2k} -z_{1k} \right) ^3 \right] \end{aligned}$$

33 $$\begin{aligned} K_{Q} = \kappa \sum _{k=1}^K \tilde{C}_{55}^k b_k \left( z_{2k} - z_{1k} \right) \end{aligned}$$

One observes that the bending stiffness $K_M$ in (32) also depends on the piezoelectric material properties. For the benchmark examples in Sect. 2.3 with the parameters from Table 3 this effect can be neglected.

For the benchmark examples presented in Sect. 3, results from 2D finite element analysis show that the shear stress distribution closely follows the parabolic distribution. Accordingly, a shear correction factor of $\kappa \approx 5/6$ is considered. Without going further into detail, for a piezoelectric multilayer beam with various elastic properties, this topic should be further investigated by considering the piezoelectric effect based on the contributions of Raman et al. [33] and Kugler et al. [34].

One observes the influence of the piezoelectric effect on the axial force and on the bending moment due to the piezoelectric coupling constants $P^k_N$ and $P^k_M$, see Eqs.(13) and (14).

The last term in Eq. (20) is the work done by the external load

34 $$\begin{aligned} \int _S p_i \delta u_i \, \textrm{d}S = \int _0^l p_x \delta u_0 + p_z \delta w_0 \, \textrm{d}x \end{aligned}$$

Combining Eqs.(21), (25), (26) and (34), one finally obtains the differential equations of the coupled transverse-axial vibrations of a piezoelectric multilayer beam

35 $$\begin{aligned} B_{uu} \ddot{u}_{0} + B_{u \psi } \ddot{\psi } - K_{N} u_{0,xx} - K_{NM} \psi _{,xx} - \sum _{k=1}^K P_{N}^k V^k_{,x} = p_x \end{aligned}$$

36 $$\begin{aligned} B_{u \psi } \ddot{u}_{0} + B_{\psi \psi } \ddot{\psi } - K_{NM} u_{0,xx} - K_{M} \psi _{,xx} - \sum _{k=1}^K P_{M}^k V^k_{,x} + K_Q \left( \psi + w_{0,x} \right) = 0 \end{aligned}$$

37 $$\begin{aligned} B_{ww} \ddot{w}_{0} - K_Q \left( \psi _{,x} + w_{0,xx} \right) = p_z \end{aligned}$$

where the following relations must be fulfilled at the boundaries

38 $$\begin{aligned} \textrm{at} \quad x = 0 \quad \textrm{and} \quad x=l: \quad N = 0 \quad \textrm{or} \quad \delta u_{0} = 0 \nonumber \\ \textrm{at} \quad x = 0 \quad \textrm{and} \quad x=l: \quad M = 0 \quad \textrm{or} \quad \delta \psi = 0 \nonumber \\ \textrm{at} \quad x = 0 \quad \textrm{and} \quad x=l: \quad Q = 0 \quad \textrm{or} \quad \delta w_{0} = 0 \end{aligned}$$

It is noted that the electrical and the mechanical domain are fully coupled by the four partial differential equations (17), (35), (36) and (37) in order to compute the deflections $u_{0}$, $w_{0}$, the rotation $\psi$ and the potential drop $V^k$. Most practical applications involving the piezoelectric effect can be divided into active applications, where the electric voltage is prescribed, and passive applications, which are used for sensing mechanical quantities (e.g structural health monitoring) and energy harvesting. In case of actuation, the voltage distribution $V^k (x)$ is usually known and only the mechanical equations (35)–(37) must be solved. In case of sensing, the voltage distribution $V^k (x)$ is not known and depends on the velocity-strain (=term on the right-hand-side in (17)).

### Vibrations of a symmetric piezoelectric Timoshenko bimorph with resistive-inductive electrodes

In the following we focus on a symmetric piezoelectric bimorph and the direction of polarization is the same for the upper and the lower piezoelectric layer. For the thickness coordinates of the lower and upper layer (subscripts *l* and *u*) $$z_{1l} = z_{2u} = 0$$ and $$z_{2l} = -z_{1l} = h_p$$ holds, where $$h_p$$ is the thickness of the piezoelectric layers, and it follows that $$K_{NM} = B_{u \psi } = 0$$ vanish, see Eqs.(22) and (30). Hence, for the piezoelectric constants follows $$P^l_{M} = -P^u_{M} = P_{M}/2 $$ and $$P^l_{N} = P^u_{N} = P_{N} $$, see Eqs.(13) and (14). Furthermore, the inner electrodes are grounded $$\varphi (x,z_{1l}) = \varphi (x,z_{2u}) = 0$$ (i.e. by keeping the inner electrodes at the same electric potential $$r_1^l = r_2^u = 0$$) and for the capacitance $$c^l = c^u = c$$ and for the external electrodes $$r_2^l = r_1^u = r$$ hold.

39 $$\begin{aligned} -V^l_{,xx} + c r \dot{ V}^l = r \left( P^l_{M} \dot{\psi }_{,x} + P^l_{N} \dot{u}_{0,x} \right) \end{aligned}$$

40 $$\begin{aligned} -V^u_{,xx} + c r \dot{ V}^u = r \left( P^u_{M} \dot{\psi }_{,x} + P^u_{N} \dot{u}_{0,x} \right) \end{aligned}$$

It is clear that in the case of bending vibrations ($u_0 = 0, \, w_0 \ne 0 \, \psi \ne 0$) the potential drop at the upper and lower piezoelectric layers must be sign-reversed $V^l = -V^u$. For the mechanical equations one derives from Eqs.(35)–(37) the simplified relation by considering $V^l = -V^u = V$

41 $$\begin{aligned} B_{\psi \psi } \ddot{\psi } - K_{M} \psi _{,xx} - P_{M} V_{,x} + K_Q \left( \psi + w_{0,x} \right) = 0 \end{aligned}$$

42 $$\begin{aligned} B_{ww} \ddot{w}_{0} - K_Q \left( \psi _{,x} + w_{0,xx} \right) = p_z \end{aligned}$$

and

43 $$\begin{aligned} -V_{,xx} + c r \dot{ V} = r \frac{ P_{M} }{ 2 } \dot{\psi }_{,x} \end{aligned}$$

Additionally, the relation between the electrode current *i*(*x*, *t*) and the voltage *V*(*x*) follows from Eq. (16): $V_{,x} = -i r$. Hence, three possibilities for the electrical boundary conditions at $x=x^*$ are possible:

44 $$\begin{aligned} V(x^*) = 0 \ldots \mathrm { both \,\, electrodes \,\, are \,\, short-circuited} \end{aligned}$$

45 $$\begin{aligned} i(x^*) = 0 \rightarrow V_{,x}(x^*) = 0 \ldots \mathrm { both \,\, electrodes \,\, are \,\, open-circuited } \end{aligned}$$

46 $$\begin{aligned} V(x^*) = i(x^*) R_{load} \rightarrow -V_{,x}(x^*) R_{load}/r = V(x^*) \ldots \mathrm { both \,\, electrodes \,\, } \nonumber \\ \mathrm {are \,\, connected \,\, via \,\, the \,\, terminal \,\, impedance\,}R_{load} \end{aligned}$$

For the sake of completeness the bending moment and shear force distribution are, see Eqs.(28) and (29)

47 $$\begin{aligned} M = K_M \psi + P_M V \qquad Q = K_Q \left( \psi + w_{0,x} \right) \end{aligned}$$

### Finite element formulation

For the derivation of the finite element formulation, the functions for $w_0$, $\psi$ and *V*(*x*) are approximated by a finite set of basis functions $\textbf{g}_{w}$, $\textbf{g}_{\psi }$, $\textbf{g}_{V}$. The vector of the nodal deflections and the rotations reads $\textbf{x} ^T = [ w_{1},\, w_{2},\, \psi _{1},\, \psi _{2} ]$ and the vector of the nodal voltages is $\textbf{V} ^T = [ V_{1},\, V_{2}$ ], where the subscript 1 and 2 refer to the deflections, rotations and voltages of the left (at $x=0$) and the right node (at $x=l$) of a finite element

48 $$\begin{aligned} {w}_{0} (x) = \textbf{g}_{w} (x)^T \textbf{x} \end{aligned}$$

49 $$\begin{aligned} {\psi } (x) = \textbf{g}_{\psi } (x)^T \textbf{x} \end{aligned}$$

50 $$\begin{aligned} {V} (x) = \textbf{g}_{V} (x)^T \textbf{V} \end{aligned}$$

The basis functions read

51 $$\begin{aligned} \textbf{g}_{w} (x) = \left[ \begin{array}{c} 1-\xi + \beta \xi \left( 1-3\xi +2\xi ^2 \right) \\ \xi - \beta \xi \left( 1-3\xi +2\xi ^2 \right) \\ -l \xi \left( 1-\xi \right) /2 - l \beta \xi \left( 1-3\xi +2\xi ^2 \right) /2 \\ l \xi \left( 1-\xi \right) /2 + l \beta \xi \left( 1-3\xi +2\xi ^2 \right) /2 \end{array} \right] \end{aligned}$$

52 $$\begin{aligned} \textbf{g}_{\psi } (x) = \left[ \begin{array}{c} 6\beta \left( 1-\xi \right) \xi /l \\ -6\beta \left( 1-\xi \right) \xi /l \\ 1-\xi - 3 \beta \xi \left( 1-\xi \right) \\ \xi - 3 \beta \xi \left( 1-\xi \right) \\ \end{array} \right] \end{aligned}$$

53 $$\begin{aligned} \textbf{g}_{V} (x) = \left[ \begin{array}{c} 1-\xi \\ \xi \end{array} \right] \end{aligned}$$

Here the non-dimensional variable $\xi = x/l$ and the parameter $\beta = ( 1 + 12 K_M/K_Q )^{-1}$ are introduced, see Knothe and Wessels [31]. Note that the Bernoulli-Euler model is obtained by assuming an infinite shear stiffness $K_Q \rightarrow \infty$, so $\beta = 1$ follows. Multiplying Eq. (41) by $\delta \psi$, Eq. (42) by $\delta w_0$ and Eq. (43) by $\delta V$ one derives the electromechanically coupled matrix equations of motion

54 $$\begin{aligned} \left[ \begin{array}{cc} \textbf{M}_{mech} &  \textbf{0} \\ -\textbf{M}_{c} &  \textbf{M}_{elec} \\ \end{array} \right] \left[ \begin{array}{c} \mathbf {\ddot{x}}\\ \mathbf {\ddot{V}}\\ \end{array} \right] + \left[ \begin{array}{cc} \textbf{0} &  \textbf{0} \\ -\textbf{C}_{c} &  \textbf{C}_{elec} \\ \end{array} \right] \left[ \begin{array}{c} \mathbf {\dot{x}}\\ \mathbf {\dot{V}}\\ \end{array} \right] + \left[ \begin{array}{cc} \textbf{K}_{mech} &  \textbf{K}_{c} \\ \textbf{0} &  \textbf{K}_{elec} \\ \end{array} \right] \left[ \begin{array}{c} \mathbf {{x}}\\ \mathbf {{V}}\\ \end{array} \right] = \left[ \begin{array}{c} \textbf{ F }\\ \textbf{ 0 }\\ \end{array} \right] \end{aligned}$$

where the matrices $\textbf{M}_{c}$, $\textbf{M}_{elec}$ are present only for inductive electrodes. The mechanical equations consist of the mass matrix $\textbf{M}_{mech}$ (with the rotary and the translational inertia)

55 $$\begin{aligned} \textbf{M}_{mech} = \int _{0}^{l} B_{\psi \psi } \textbf{g}_{\psi } \textbf{g}_{\psi }^T + B_{ww} \textbf{g}_{w} \textbf{g}_{w} ^T \, \textrm{d}x \end{aligned}$$

the stiffness matrix $\textbf{K}_{mech}$ (with the bending and the shear stiffness)

56 $$\begin{aligned} \textbf{K}_{mech} = \int _{0}^{l} K_M \textbf{g}_{\psi ,x} \textbf{g}_{\psi ,x} ^T + K_Q \left( \textbf{g}_{\psi } + \textbf{g}_{w,x} \right) \left( \textbf{g}_{\psi } + \textbf{g}_{w,x} \right) ^T \, \textrm{d}x \end{aligned}$$

and the load vector $\textbf{F}$

57 $$\begin{aligned} \textbf{F} = \int _{0}^{l} p_z \textbf{g}_{w} \, \textrm{d}x + \left[ F_1, \, M_1,\, F_2,\, M_2 \right] ^T \end{aligned}$$

where $F_1, \, F_2$ are the single forces and $M_1, \, M_2$ are the moments at the left and right nodes.

The electrical equations consist of the electrical inertia $\textbf{M}_{elec}$, which depends on the electrode inductance *l*, of the electrical capacity $\textbf{C}_{elec}$, which depends on the electrode resistance *r*, and of the electrical stiffness $\textbf{K}_{elec}$

58 $$\begin{aligned} \textbf{M}_{elec} = l c \int _{0}^{l} \textbf{g}_{V} \textbf{g}_{V}^T \, \textrm{d}x \end{aligned}$$

59 $$\begin{aligned} \textbf{K}_{elec} = \int _{0}^{l} \textbf{g}_{V,x} \textbf{g}_{V,x} ^T \, \textrm{d}x \end{aligned}$$

60 $$\begin{aligned} \textbf{C}_{elec} = r c \int _{0}^{l} \textbf{g}_{V} \textbf{g}_{V}^T \, \textrm{d}x \end{aligned}$$

The mechanical and electrical domains (54) are coupled by

61 $$\begin{aligned} \textbf{K}_{c} = P_M \int _{0}^{l} \textbf{g}_{\psi ,x} \textbf{g}_{V} ^T \, \textrm{d}x \end{aligned}$$

62 $$\begin{aligned} \textbf{C}_{c} = r \frac{ P_M }{2} \int _{0}^{l} \textbf{g}_{V} \textbf{g}_{\psi ,x}^T \, \textrm{d}x \end{aligned}$$

63 $$\begin{aligned} \textbf{M}_{c} = l \frac{ P_M }{2} \int _{0}^{l} \textbf{g}_{V} \textbf{g}_{\psi ,x}^T \, \textrm{d}x \end{aligned}$$

One observes that from Eqs.(61) and (62) it follows that $\textbf{K}_{c} r/2 = \textbf{C}_{c}^T$ .

## Numerical benchmark example – Validation to finite element results

In this section, the one-dimensional piezoelectric beam model with Timoshenko kinematics is compared to two-dimensional finite element results. A thin beam is investigated with thickness-to-length ratio $\lambda = 1/40$ (Sect. 3.1) before results for the moderately thick beam are compared ($\lambda = 1/10$, Sect. 3.2). The same benchmark example as in a previous contribution (Schoeftner et al. [29]) is considered: A clamped-hinged symmetric piezoelectric bimorph is considered, hence $w(0) = w(l) = 0$ and $\psi (0) = 0$ for the mechanical boundary conditions. The beam is subjected to the sinusoidal distributed load $p_z(x,t) = 25 \cdot \cos \omega t$. The geometric dimensions and the material parameters are listed in Table 3. The length of the beam is $l=0.04 \textrm{m}$ and the width is $b_p = 0.004 \textrm{m}$. The thickness of the piezoelectric layers is $h_p = 5 \times 10^{-4} \textrm{m}$ for the thin beam (the thickness-to-length ratio is $\lambda =1/40$) and $h_p = 20 \times 10^{-4} \textrm{m}$ for the moderately thick beam (the thickness-to-length ratio is $\lambda =1/10$). For the electrical boundaries, it is assumed that the electrodes are open-circuited at $x=0$ (i.e. $V_{,x}(0,t) = 0$, see Eq. (45)). At $x=l$, an electrical resistance $R_{load}$ is linked between both ends, see Eq. (46).

The finite element (FE) code is written in MATLAB where a Q8 element (eight-node quadrilateral element with quadratic shape functions for both displacement and voltage degrees of freedom) is used. The modified FE code is based on the book by Ferreira [35], who presents a wide variety of standard finite elements (beams, plates and two-dimensional elements) for elastic structures. Ferreira’s original code is adapted for this research and piezoelectric properties are properly taken into account, see Piefort [36]. For the thin piezoelectric bimorph with $\lambda =1/40$ (Sect. 3.1), the number of elements in the *x*- and *z*-directions are $n_x \times n_z = 208 \times 14 = 2912$, yielding a total number of 9181 nodes for the rectangular Q8 mesh and an element aspect ratio $AR=2.69$. For the piezoelectric bimorph with $\lambda =1/10$ (Sect. 3.2), the number of elements is $n_x \times n_z = 112 \times 26 = 2912$, hence the number of nodes is 9013 and $AR=2.32$. A parametric study of the number of elements and the aspect ratio showed that further mesh refinements do not significantly change the results and that convergence is achieved. This MATLAB FE-code was used for validation in previous contributions by the author concerning refined elastic and piezoelectric beam theories, see Schoeftner [32] or [37].

In order to secure the zero voltage condition over the inner electrodes, the electrical degrees of freedom are kept at zero potential.

### Thin beam ($\lambda = 1/40$)

#### Eigenfrequencies

First the eigenfrequencies of the three finite element models (1D FE–a piezoelectric Bernoulli-Euler beam, 1D TS–a piezoelectric Timosheko beam beam, 2D FE–two-dimensional piezoelectric continuum model) are compared for the thin beam with $h_p = 0.5 \textrm{mm}$, see Table 1.

**Table 1:** Natural frequencies (Hz) of a clamped-hinged beam ($\lambda =1/40$)

Variable (unit)	$f_1$	$f_2$	$f_3$	$f_4$
Short circuit (1D BE)	1260.6	4086.6	8538.0	$14 \, 647.4$
Open circuit (1D BE)	1266.0	4091.8	8543.2	$14 \, 652.4$
Non-electroded (1D BE)	1299.4	4212.4	8800.2	$15 \, 094.2$
Short circuit (1D TS)	1257.0	4056.6	8423.0	$14 \, 349.1$
Open circuit (1D TS)	1262.4	4061.6	8428.2	$14 \, 352.0$
Non-electroded (1D TS)	1295.5	4179.8	8675.8	$14 \, 769.0$
Short circuit (2D FE)	1258.3	4059.6	8415.1	$14 \, 269.4$
Open circuit (2D FE)	1263.8	4064.4	8420.3	$14 \, 279.0$
Non-electroded (2D FE)	1296.1	4178.7	8654.0	$14 \, 657.3$

Abbreviations: 1D BE$\ldots$one-dimensional Bernoulli-Euler finite element results 1D TS$\ldots$one-dimensional Timoshenko finite element results 2D FE$\ldots$two-dimensional finite element (Q8) results under plane stress conditions

As expected, the results for the short-circuited (sc) eigenfrequencies are always lower than the open-circuited (oc) eigenfrequencies. The highest ones are obtained by the non-electroded (ne) configuration ($f_{sc}< f_{oc} < f_{ne}$). The first eigenfrequencies are almost identical for the Bernoulli, the Timoshenko and the 2D-beam mode, but one observes that only the Timoshenko beam frequencies are very close to the 2D beam results even for the third and fourth eigenfrequencies ($f_{3sc} = 8423 \, \textrm{Hz}$ (TS) $\approx$ $f_{3sc} = 8415 \, \textrm{Hz}$ (2D)). Both open-circuited eigenfrequencies are $5.2 \, \textrm{Hz}$ larger than the short-circuited ones. The fourth eigenfrequencies for the non-electroded case are $f_{4ne} = 14769 \, \textrm{Hz}$ (TS) $\approx$ $f_{4ne} = 14657.3 \, \textrm{Hz}$ (2D), so the error is still below $1\%$. The Bernoulli result overestimates the outcome by $3\%$.

#### Quasi-static deflection

**Fig. 2:**
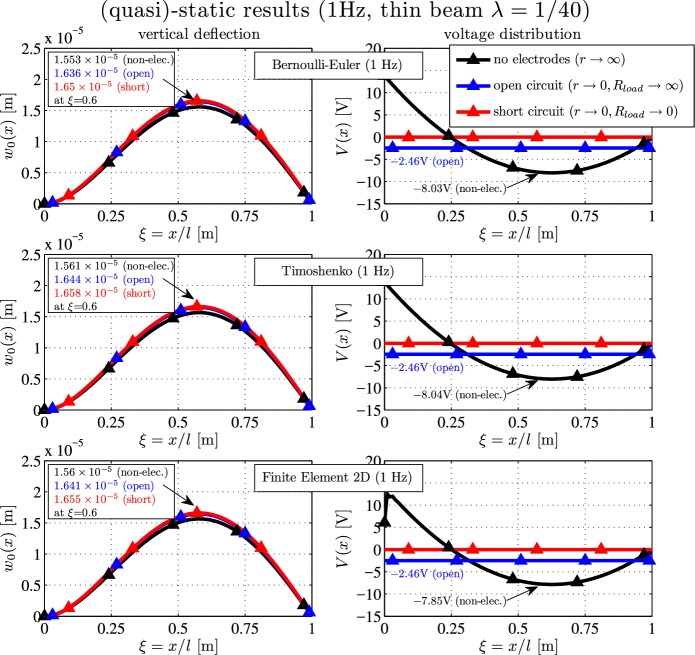
(Quasi-)static deflection $w_0(x)$ (left) and voltage distribution *V*(*x*) (right) of a thin ($\lambda = 1/40$) clamped-hinged piezoelectric bimorph for various electrical boundary conditions

The quasi-static deflections and the voltage distribution at $f=1 \, \textrm{Hz}$ (which is much lower than the first eigenfrequency) are shown in Figure 2.

The configuration with the largest deflection is the short-circuit one (red), while the stiffest configuration is the non-electroded case (black). The maximum deflection values are shown in the small boxes in the displacement plot. One observes that Bernoulli, Timoshenko and the 2D results are in good agreement: the maximum open-circuit results read $1.636 \times 10^{-5} \, \textrm{m}$ (BE), $1.644 \times 10^{-5} \, \textrm{m}$ (TS) and $1.641 \times 10^{-5} \, \textrm{m}$ (2D). Although the differences are very small, the Timoshenko results are also closer to the target solutions (2D FE) for the other electrical boundary conditions. The open-circuit voltage is $-2.46 \, \textrm{V}$ for all three finite element models. The lowest value for the voltage distribution (no electrodes) occurs at $\xi \approx 0.63$ and reads $-8.03 \, \textrm{V}$ (BE), $-8.04 \, \textrm{V}$ (TS) and $-7.85 \, \textrm{V}$ (2d FE).

#### Passive damping by optimal resistances and inductances

In this subsection, we focus on the practical relevance of the optimal resistive-inductive properties of the electrodes and the terminating resistances and inductances. It is shown that resistive electrodes might attenuate structural vibrations much more efficiently than an attached resistive circuit. Adding inductive properties and optimal parameter tuning might further increase the damping capabilities. These theoretical investigations might be useful for practical experimental setups in the future. The following six parameter combinations for optimal resistive and resistive-inductive passive vibration control are considered:

- case (a): optimal terminal resistance and perfect electrodes: $r = 0 \, \Omega /\textrm{m}$, $R_{load} = 18 \, 744 \, \Omega$
- case (b): optimal resistive electrodes with short-circuited electrodes: $r = 11.34 \times 10^{6} \, \Omega /\textrm{m}$, $R_{load} = 0 \, \Omega$
- case (c): optimal resistive electrodes with open-circuited electrodes: $r = 9.97 \times 10^{6} \, \Omega /\textrm{m}$, $R_{load} \rightarrow \infty$
- case (d): optimal resistive-inductive load and perfect electrodes: $r = 0 \, \Omega /\textrm{m}$, $l = 0 \, \mathrm {H/m}$, $R_{load} = 2328 \, \Omega$, $L_{load} = 2.33 \, \textrm{H}$
- case (e): optimal resistive-inductive electrodes with short-circuited electrodes: $r = 2615.4 \times 10^{3} \, \Omega /\textrm{m}$, $l = 1283.8 \, \mathrm {H/m}$, $R_{load} = 0 \Omega$, $L_{load} = 0 \, \textrm{H}$
- case (f): optimal resistive-inductive electrodes with open-circuited electrodes: $r = 846 \times 10^{3} \, \Omega /\textrm{m}$, $l = 566 \, \mathrm {H/m}$, $R_{load} \rightarrow \infty$

**Fig. 3:**
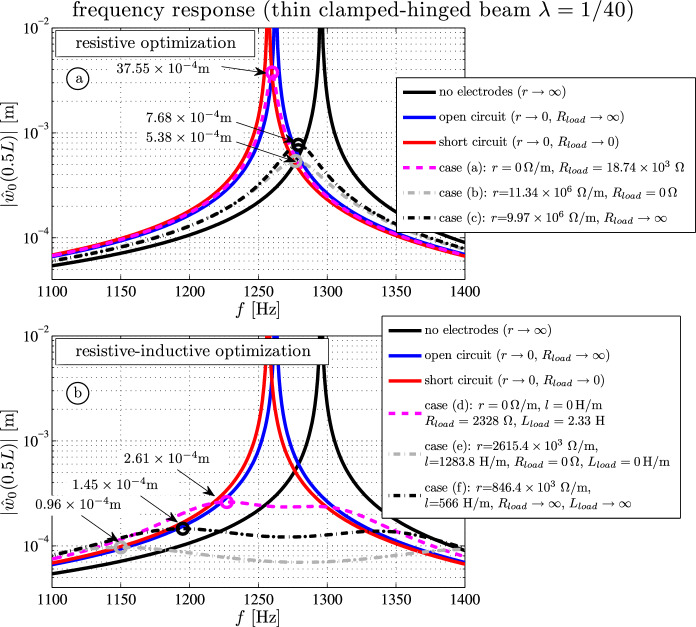
Frequency response $|\hat{w}_0(l/2)|$ for the Timoshenko beam around the first eigenfrequency for short-circuit, open-circuit, non-electroded boundary conditions and optimal resistive and resistive-inductive values of *r*, *l*, $R_{load}$ and $L_{load}$ (cases **a**–**f**)

Figure 3 shows the frequency responses for the deflection close to the first natural frequency. As a reference, one observes the undamped peaks for the non-electroded (black, at $1295.5\,\textrm{Hz}$), the open-circuited (blue, at $1262.4\,\textrm{Hz}$) and the short-circuited (red, at $1257\,\textrm{Hz}$) cases. If only the terminating resistor is optimized, the maximum deflection is $37.55 \times 10^{-4}\, \textrm{m}$ (Figure 3a), which corresponds to a modal damping of $d=0.19 \%$ (i.e. the first mode of a short- or open-circuited beam with a modal damping value $d=0.19$ would exhibit the same peak amplitude as the electrically damped beam).

Hagood and Flotow [8] and Park [10] suggest $R_{load} = 1/( 2\pi f C_p)$ as the optimal resistor value. This yields $R_{load} \approx 18744 \, \Omega$ (case (a)–magenta dash-dotted) where the piezoelectric capacitance $C_p \approx 168 \times 10^{-9}\,\mathrm {As/V}$ is the product of sheet capacitance *c* and the length of the beam.

If the terminating resistor is zero (i.e. short-circuited electrodes at the right side $V(l) = 0$) and the sheet resistance is $r = 11.34 \times 10^{6} \, \Omega /\textrm{m}$ the maximal deflection is only one-seventh: $5.38 \times 10^{-4}\, \textrm{m}$ (case (b)–grey dash-dotted). If the electrodes are open-circuited at the right side $i(l) = 0$, the deflection is slightly higher: $7.68 \times 10^{-4}\, \textrm{m}$ (case (c)–black dash-dotted). The equivalent modal damping for these two cases are $d=1.1 \%$ (case (c)) and $d=1.8 \%$ (case (b)), respectively. The mechanical damping for metals ranges from $1\%$ to $3\%$. From a practical point of view, it follows that only resistive electrodes reduce the peak values (cases (b) and (c)). This does not hold for an attached resistive circuit (case (a)).

Figure 3b shows the results for resistive-inductive electrodes and load impedances.

The optimal value for the inductor *L* (case (d)) is suggested by Park [10] as $\delta = 1/\sqrt{L C_p \omega ^2}$, where $\delta$ is the non-dimensional tuning ratio for which the electrical resonant frequency is tuned in the vicinity of a mechanical resonant frequency. For the cases (e) and  (f), the best values of the electrode conductivity are determined by trial and error.

While the $RL_{load}$-circuit has double peaks with $2.61 \times 10^{-4}\, \textrm{m}$ at $f \approx 1230 \, \textrm{Hz}$ and $f \approx 1300 \, \textrm{Hz}$ (case (d)–magenta dash-dotted), the highest deflections for *rl*-optimized electrodes with either short- or open-circuited electrodes at the right side are $1.45 \times 10^{-4}\, \textrm{m}$ (case (e)–grey dash-dotted) or $0.96 \times 10^{-4}\, \textrm{m}$ (case (f)–black dash-dotted).

These results demonstrate that the damping effects of optimized electrodes may significantly reduce structural vibrations compared to optimized terminal impedances. In practical applications resistive electrodes may be realized by several piezoelectric patches attached onto the substrate with electric circuits connecting the patch electrodes (see Figure 1a and b).

### Moderately thick beam ($\lambda = 1/10$)

### Eigenfrequencies

The eigenfrequencies of the three finite element models (1D FE BE, 1D TS, 2D FE) are compared for a beam with $h_p = 2 \textrm{mm}$, see Table 2. Unlike the thin beam results in Table 1, one observes that the Bernoulli-Euler result overestimates the first short-circuited mode by $3.7\%$ ($f_{1sc} = 5042.5 \, \textrm{Hz}$ (BE)), while the 2D FE results is $f_{1sc} = 4856.2 \, \textrm{Hz}$. The Timoshenko result is very close to the two-dimensional finite element results $f_{1sc} = 4831.2 \, \textrm{Hz}$ (TS), which has a relative error of $-0.5\%$ only. For the higher modes, the Bernoulli-Euler model severely overestimates the eigenfrequencies, only the Timoshenko beam yields reliable results (despite using only 10 finite elements for the 1D model), cf. $f_{4oc} = 28635.4 \, \textrm{Hz}$ (TS) and $f_{4oc} = 28699.7 \, \textrm{Hz}$ (2D FE). It can be seen that the differences between short-circuited and open-circuited results are low, the results for the non-electroded configuration yield the highest eigenfrequencies.

It is noted that the third column in Table 2 shows the results for the first longitudinal mode, which cannot be predicted with the discretized beam model Eqs.(41) and (42). For this purpose the more general, coupled axial-bending equations (35)–(37) must be solved.

**Table 2:** Natural frequencies (Hz) of a clamped-hinged beam ($\lambda =1/10$)

Variable (unit)	$f_1$	$f_2$	$f_3$	$f_4$	$f_5$
Short circuit (1D BE)	5042.5	$16 \, 349.0$	(longitudinal mode)	$34 \, 152.1$	58589.4
Open circuit (1D BE)	5064.0	$16 \, 366.9$	(longitudinal mode)	$34 \, 172.9$	58609.6
Non-electroded (1D BE)	5197.5	$16 \, 848.5$	(longitudinal mode)	$35 \, 200.8$	60376.6
Short circuit (1D TS)	4831.2	$14 \, 716.8$	(longitudinal mode)	$28 \, 621.4$	$45 \, 765.8$
Open circuit (1D TS)	4851.2	$14 \, 733.0$	(longitudinal mode)	$28 \, 635.4$	$45 \, 777.8$
Non-electroded (1D TS)	4968.6	$15 \, 093.6$	(longitudinal mode)	$29 \, 271.6$	$46 \, 683.8$
Short circuit (2D FE)	4856.2	$14 \, 814.9$	$17 \, 654.0$	$28 \, 685.9$	$45 \, 253.7$
Open circuit (2D FE)	4877.0	$14 \, 825.2$	$17 \, 654.0$	$28 \, 699.7$	$45 \, 262.7$
Non-electroded (2D FE)	4974.1	$15 \, 080.6$	$18 \, 364.1$	$29 \, 025.3$	$45 \, 588.8$

Abbreviations: 1D BE$\ldots$one-dimensional Bernoulli-Euler finite element results 1D TS$\ldots$one-dimensional Timoshenko finite element results 2D FE$\ldots$two-dimensional finite element (Q8) results under plane stress conditions

#### Quasi-static deflection

**Fig. 4:**
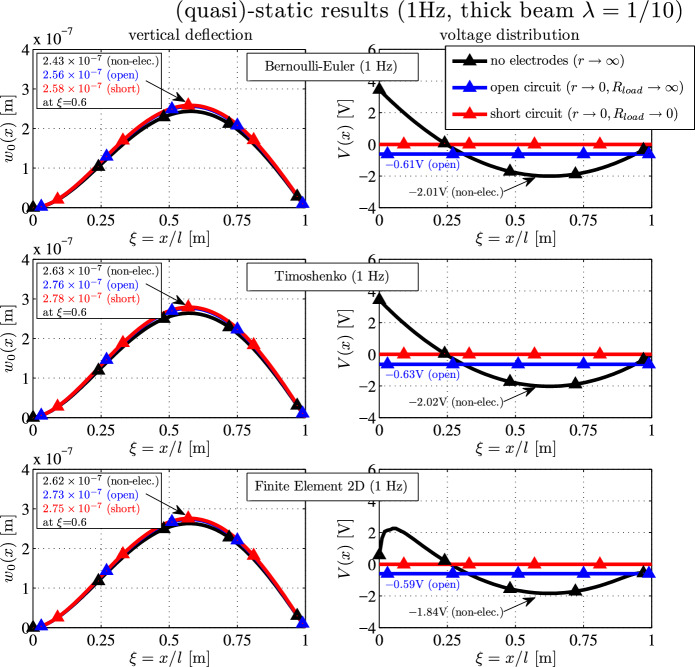
(Quasi-)static deflection $w_0(x)$ (left) and voltage distribution *V*(*x*) (right) of a moderately thick ($\lambda = 1/10$) clamped-hinged piezoelectric bimorph for various electrical boundary conditions

Finally, the quasi-static deflections and the voltage distribution at $f=1 \, \textrm{Hz}$ are shown for the beam with $\lambda = 1/10$ in Figure 4. The short-circuited deflections (red) yield $2.58 \times 10^{-7} \, \textrm{m}$ (BE), $2.78 \times 10^{-7} \, \textrm{m}$ (TS) and $2.75 \times 10^{-7} \, \textrm{m}$ (2D-FE), respectively. The Bernoulli beam is again much too stiff ($-6.5\%$ error), but the Timoshenko beam has an error of only $1\%$. The open-circuited results are slightly lower than the short-circuited results. For the non-electroded configuration, the Bernoulli model underestimates the result by $-7 \%$ ($w_{0 max} \approx 2.43 \times 10^{-7} \textrm{m}$), while the maximum Timoshenko and the 2D-FE beam deflections are $2.62 \times 10^{-7} \textrm{m}$.

The qualitative shape of the non-electroded voltage distribution is the same for all three configurations, but both 1D-models underestimate the target outcome $-2.01 \, \textrm{V}< -1.84 \, \textrm{V}$. This may be suggest that for sensing applications, the electric field and the electric displacement in the *x*-direction may not be disregarded, see e.g. Krommer [15, 16] or Schoeftner [37].

## Conclusion

In this contribution, a one-dimensional theory for smart piezoelectric beams with Timoshenko kinematics and finitely conductive electrodes is derived. Contrary to the current state of the art, where the equipotential area condition over the electrodes is assumed, the electromechanical coupling of imperfect electrodes with mechanical deformations is investigated. The outcomes of the extended piezoelectric beam theory are four differential equations, coupling mechanical displacements (axial and transverse deflection, rotation angle) to the electric potential. The derived one-dimensional piezoelectric Timoshenko equations are compared to two-dimensional electromechanically coupled finite element results. A thin and a moderately thick clamped-hinged piezoelectric bimorph are considered. Natural frequencies, static deflections, and harmonic responses of the vertical deflection and the voltage distributions are compared between one-dimensional finite element results (based on piezoelectric Bernoulli-Euler and Timoshenko beams) and two-dimensional finite element results. It is shown that results are in very good agreement for the Timoshenko beam and the 2D finite element results, even for the thick beam as well as for the higher eigenmodes. Finally, the practical relevance of conductive electrodes is pointed out. It is shown that resistive-inductive electrodes have a great potential for attenuating vibrations for passive vibration control: vibrations are damped much more efficiently compared to resistive electrodes or to optimally attached resistive-inductive circuits.

## Appendix A Material properties PZT-5A

The three-dimensional constitutive relations read

A1 $$\begin{aligned} \begin{array}{cccccc} {\varvec{\sigma = C \epsilon -e}}^T {\varvec{E}} \\ {\varvec{D= e \epsilon +\kappa E}.} \end{array} \end{aligned}$$

Assuming plane stress in the *xz*-plane and a vanishing out-of plane electric displacement component

A2 $$\begin{aligned} \sigma _{yy}=\sigma _{xy}=\sigma _{yz}= D_y=0, \end{aligned}$$

the constitutive components are rearranged to

A3 $$\begin{aligned} \begin{array}{rclrclrclrclrcl} \bar{C}_{11} & =&  {C}_{11}-\frac{{C}_{12}^2}{{C}_{22}} \quad &  \bar{C}_{13} & =&  {C}_{13}-\frac{{C}_{12} {C}_{23} }{{C}_{22}} \quad &  \bar{C}_{33} & =&  {C}_{33}-\frac{{C}_{23}^2 }{{C}_{22}} &  \\ \\ \bar{C}_{55} & =&  {C}_{55} \quad &  \bar{e}_{31} & =&  {e}_{31}- {e}_{32}\frac{{C}_{12}}{{C}_{22}} \quad &  \bar{e}_{33} & =&  {e}_{33}- {e}_{32}\frac{{C}_{23}}{{C}_{22}} \quad &  \\ \\ \bar{e}_{15} & =&  {e}_{15} &  \bar{\kappa }_{11} & =&  {\kappa }_{11} \quad &  \bar{\kappa }_{33} & =&  {\kappa }_{33}+\frac{e_{32}^2}{C_{22}}. &  \end{array} \end{aligned}$$

For beam-like structures, we additionally demand the thickness component of the normal stress to be negligible and the axial component of the electric field vector to be zero

A4 $$\begin{aligned} \sigma _{zz}={E_x} = 0. \end{aligned}$$

Hence, the following equivalent one-dimensional effective values for piezoelasticity used in our analytical calculation are obtained as

A5 $$\begin{aligned} \begin{array}{rclrclrclrclrcl} \tilde{C}_{11} & =&  \bar{C}_{11} -\frac{\bar{C}_{13}^2}{\bar{C}_{33}} &  \quad \tilde{C}_{55} & =&  \bar{C}_{55} \\ \\ \tilde{e}_{31} & =&  \bar{e}_{31} - \bar{e}_{33} \frac{\bar{C}_{13}}{\bar{C}_{33}} &  \quad \tilde{e}_{15} & =&  \bar{e}_{15} &  \quad \tilde{\kappa }_{33} & =&  \bar{\kappa }_{33} + \frac{\bar{e}_{33}^2}{\bar{C}_{33}}. \end{array} \end{aligned}$$

The material properties for the piezoelectric transducers used in this study read:

- Density ($$\textrm{kgm}^{-3} $$): $$\rho =7750$$
- Elasticity components in Voigt notation ($\textrm{Nm}^{-2}$): $C_{11}=C_{22}=121.00\times10^{9},\,C_{12}=75.40\times10^{9},\,C_{13}=C_{23}=75.20\times10^{9},\,C_{33}=111.00\times10^{9},\,C_{44}=C_{55}=21.10\times10^{9},\,{C_{66}=\frac{1}{2} \left(C_{11}-C_{12}\right)=22.80 \times 10^{9}}$, remaining components $C_{ij}=0$
- Piezoelectric components in Voigt notation ($\textrm{Cm}^{-2}$): $e_{31}=e_{32}=-5.40,\, e_{33}=15.80, \,$ $e_{24}=e_{15}=12.30$, remaining components $e_{ij}=0$
- Permittivity components in Voigt notation: $\kappa _{11}=\kappa _{22}=1649 \, \epsilon _{0},\, \kappa _{33}=1750 \, \epsilon _{0}, \epsilon _{0}=8.854 \times 10^{-12} \, \textrm{CV}^{-1}\textrm{m}^{-1}$, remaining components $\kappa _{ij}=0$

Based on these values, the effective constants on beam level (reduced axial stiffness $\tilde{C}^p_{11}, \, \tilde{C}^p_{55}$, stress piezoelectric coupling constant $\tilde{e}^p_{31}$, and blocked strain-free permittivity $\tilde{\kappa }^p_{33}$) are computed from Eqs.(A3)–(A5). Note that the superscript *p* for piezoelectric layer is introduced.

## Data for the benchmark example

Further data for the numerical example can be found in Table 3.

**Table 3:** Parameters for the numerical example

Variable (unit)	Value	Description
$\rho \,\quad (\textrm{kg m}^{-3})$	7750	Density
$\{ z_{1p},\, z_{2p} \} \,\quad (\textrm{m})$	$\{ 0,\, 5.00 \cdot 10^{-4} \}$	Thickness direction coordinates
		(Thin beam $$\lambda =1/40$$)
$\{ z_{1p},\, z_{2p} \} \,\quad (\textrm{m})$	$\{ 0,\, 2.00 \cdot 10^{-3} \}$	Thickness direction coordinates
		(Moderately thick beam $\lambda =1/10$)
$b_{p} \,\quad (\textrm{m})$	$4.00 \times 10^{-3}$	Width of piezoelectric transducer
$l \,\quad (\textrm{m})$	$4.00 \times 10^{-2}$	Beam length
$$ \tilde{e}^p_{31} \,\quad (\textrm{As m}^{-2})$$	$$-10.48 $$	Piezoelectric coupling constant, see Eqs.(A3)–(A5)
$\tilde{\kappa }^p_{33} \,\quad (\textrm{As V}^{-1} \textrm{m}^{-1})$	$2.10 \times 10^{-8}$	Permittivity, see Eqs.(A3)–(A5)
$\tilde{C}^p_{11} \,\quad (\textrm{Nm}^{-2})$	$6.15 \times 10^{10}$	Young’s modulus, see Eqs.(A3)–(A5)
$\tilde{C}^p_{55} \,\quad (\textrm{Nm}^{-2})$	$2.11 \times 10^{10}$	Shear modulus , see Eqs.(A3)–(A5)
$p_z \,\quad (\textrm{Nm}^{-1})$	25	Distributed load per unit length
